# CB2 receptor deletion on myeloid cells enhanced mechanical allodynia in a mouse model of neuropathic pain

**DOI:** 10.1038/s41598-019-43858-4

**Published:** 2019-05-16

**Authors:** Elisa Nent, Chihiro Nozaki, Anne-Caroline Schmöle, David Otte, Andreas Zimmer

**Affiliations:** Institute of Molecular Psychiatry, University of Bonn, 53127 Bonn, Germany

**Keywords:** Neuroscience, Diseases of the nervous system

## Abstract

Neuropathic pain can develop after nerve injury, leading to a chronic condition with spontaneous pain and hyperalgesia. Pain is typically restricted to the side of the injured nerve, but may occasionally spread to the contralateral side, a condition that is often referred to as mirror-image pain. Mechanisms leading to mirror-image pain are not completely understood, but cannabinoid CB2 receptors have been implicated. In this study, we use genetic mouse models to address the question if CB2 receptors on neurons or on microglia/macrophages are involved. First, we show that a GFP reporter protein under control of the CB2 promoter is induced upon partial sciatic nerve ligation in spinal cord, dorsal root ganglia, and highest in sciatic nerve macrophages, but not in neurons. Mice which lack CB2 receptors specifically on myeloid cells (microglia, macrophages) developed a mirror-image allodynia [*treatment* F_1,48_ = 45.69, p < 0.0001] similar to constitutive CB2 receptor knockout mice [*treatment* F_1,70_ = 92.41, p < 0.0001]. Such a phenotype was not observed after the deletion of CB2 from neurons [*treatment* F_1,70_ = 0.1315, p = 0.7180]. This behavioral pain phenotype was accompanied by an increased staining of microglia in the dorsal horn of the spinal cord, as evidenced by an enhanced Iba 1 expression [CB2KO, p = 0.0175; CB2-LysM, p = 0.0425]. Similarly, myeloid-selective knockouts showed an increased expression of the leptin receptor in the injured ipsilateral sciatic nerve, thus further supporting the notion that leptin signaling contributes to the increased neuropathic pain responses of CB2 receptor knockout mice. We conclude that CB2 receptors on microglia and macrophages, but not on neurons, modulate neuropathic pain responses.

## Introduction

Preparations of *Cannabis sativa* have been used for millennia to treat various pain conditions. One of the active ingredients of cannabis, ∆^9^-tetrahydrocannabinol (THC), can bind and activate the G-protein coupled cannabinoid receptor 1 (CB1)^1^ and cannabinoid receptor 2 (CB2)^2^, which are the two main receptors of the endocannabinoid system. Whereas CB1 is most abundantly expressed in the brain, CB2 is mainly found on immune cells, including macrophages and microglia^3^. CB2 expression in neurons is low under normal conditions^4–6^, but it is an important modulator of neuronal physiology and neuronal network activity^7^. Although neuronal expression of CB2 is low in healthy tissues, CB2 protein and mRNA levels are increased in DRG and spinal cord neurons under neuropathic pain conditions^8–12^. The induction of CB2 is probably part of the recuperative process, as activation of CB2 by natural and synthetic agonists reduced pain symptoms^12–25^. Genetically enhanced CB2 signaling in mice overexpressing CB2 receptors in microglia and neurons also reduced the manifestation of neuropathic pain symptoms^26^. Conversely, the deletion of CB2 resulted in an enhanced neuroinflammatory response after sciatic nerve injury and in mirror-image pain, as evidenced by the development of tactile allodynia on the contralateral side of the injured nerve^26^.

In most cases, neuropathic pain is restricted to the body region that is innervated by the affected nerve, but some animal models and human pain conditions with mirror-image pain have been described^27,28^. CB2 knockout (CB2KO) mice are one of very few genetic mouse models in which such a contralateral pain due to unilateral injury often develops, thus indicating that CB2 signaling is required to restrict the development of allodynia to the ipsilateral site. The mechanisms involved in this process nevertheless remain largely obscure, although peripheral and central inflammatory processes have been implicated^29,30^. Previously, it was shown that leptin signaling promotes neuropathic pain via modulating CB2 signaling. Mice with a deletion of the CB2 receptor showed an increase in leptin receptor expression on the ipsi- and contralateral sciatic nerve. After perineural administration of a neutralizing anti-leptin antibody, increased leptin receptor expression and tactile hyperalgesia were reduced again on both sides, ipsi- and contralateral. A contribution of leptin in neuropathic pain development through CB2 was therefore concluded^31^.

Because CB2 receptors are expressed on neurons, as well as macrophages and microglia after sciatic nerve injury, it is not clear on which cells CB2 is acting during neuropathic pain. In this study, we use CB2-GFP reporter mice and mice with a cell-specific CB2 deletion on neurons or myeloid cells to address the question, which CB2- expressing cell type is important for the development of neuropathic pain.

## Results

### CB2KO and CB2-LysM mice develop similar mechanical allodynia

**Figure 1:**
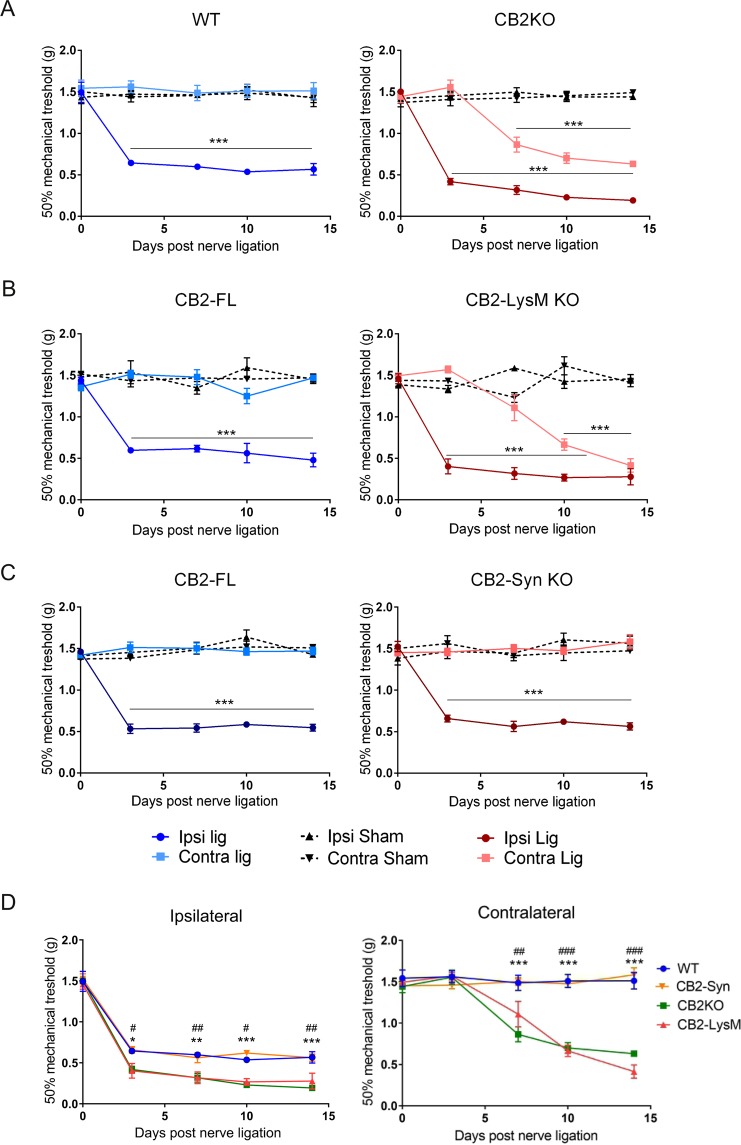
Mechanical allodynia of WT and CB2KO (**A**), CB2-LysM (**B**) and CB2-Syn (**C**) after nerve ligation. Allodynia was assessed with von Frey filaments, basal and during 14 days post SNL in ipsi- and contralateral hind paws. Ligated CB2KO and CB2-LysM mice showed increased contralateral allodynia compared to ligated WT or CB2-FL animals. Additionally, ligated CB2KO and CB2-LysM mice developed increased ipsilateral allodynia. Ligated WT and CB2-Syn mice showed a similar development of mechanical allodynia. When compared between ligated genotypes (**D**), mechanical allodynia of CB2KO and CB2-LysM animals was significantly increased on both sides, compared to WT animals (n = 4–8). Statistical significance was determined with a two-way ANOVA and a Bonferroni post-hoc test (**A**–**C**) or a Dunnet’s post-hoc test (**D**). Stars represent differences between ligated and sham animals (**A**–**C**) or between ligated CB2KO and ligated WT animals (**D**). *p < 0.05, **p < 0.01, ***p < 0.001. Hashtags indicate significance between CB2-LysM and WT mice. ^#^p < 0.05, ^##^p < 0.01, ^###^p < 0.001. Error bars show SEM.

We used cell-selective CB2 knockout mouse lines in order to assess if CB2 receptor expression on neurons or myeloid cells, or both, is relevant for neuropathic pain development. Thus, we compared SNL-induced mechanical allodynia in mice lacking CB2 receptors on neurons (CB2-Syn), or in myeloid cells (CB2-LysM), or constitutively in all cells (CB2KO). Three days after SNL all mouse strains showed mechanical allodynia in the hind paw ipsilateral to the nerve injury (Fig. 1), but not on the contralateral hind paw.

A detailed analysis of the mechanical pain responses during the whole course of the experiment revealed important genotype differences: WT mice displayed a clear *treatment* effect [F_1,60_ = 292.1, p < 0.0001] and a *treatment* × *time* interaction [F_4,60_ = 21.81, p < 0.0001]. Mechanical pain thresholds of ligated WT mice decreased on day 3 and stayed constant until day 14. Ligated CB2KO mice also developed an increased mechanical allodynia on the ipsilateral side that lasted for at least 14 days (Fig. 1A) [*treatment* F_1,70_ = 1063, p < 0.0001]. Additionally, the contralateral hind paw of ligated CB2KO mice displayed signs of neuropathic pain as well, beginning on day 7 [*treatment* F_1,70_ = 92.41, p < 0.0001]. Both sides displayed significant *treatment* × *time* interaction [ipsi: F_4,70_ = 80.45, p < 0.0001; contra: F_4,70_ = 26.56, p < 0.0001].

In contrast, ligated WT animals did not show signs of contralateral mechanical allodynia at any time point [F_1,60_ = 1.114, p = 0.2955] and sham treated animals did not develop any mechanical allodynia. Thus, as reported previously, CB2KO mice developed a delayed mirror-image pain phenotype that was not present in WT animals.

Interestingly, in CB2-LysM mice the development of mechanical allodynia was almost identical to that of CB2KO animals (Fig. 1B). We observed an increased ipsilateral mechanical allodynia starting on day 3 after SNL that was constant until day 14. A pronounced *treatment* effect was revealed for the ipsilateral hind paw [CB2-LysM: F_1,48_ = 427.9, p < 0.0001; CB2-FL: F_1,48_ = 249.7, p < 0.0001]. The contralateral side developed a similar pain response as seen in CB2KO mice, starting on day 7 after nerve ligation until day 14 [*treatment* F_1,48_ = 45.69, p < 0.0001]. Both sides showed a significant *time* × *treatment* effect [ipsi: F_4,48_ = 41.46, p < 0.0001; contra: F_4,48_ = 20.42, p < 0.0001]. Again, mechanical allodynia in Cre-negative control littermates with a “floxed” CB2 gene locus (CB2-FL) only developed on the ipsilateral side [*treatment* F_1,48_ = 249.7, p < 0.0001] and remained on basal levels on the contralateral side [*treatment* F_1,48_ = 2.225, p = 0.1423].

In CB2-Syn mice (Fig. 1C), mechanical allodynia was detected only on the ipsilateral side, similar as in ligated CB2-FL control littermates [*treatment* CB2-Syn: F_1,70_ = 294.3, p < 0.0001; CB2-FL: F_1,70_ = 534.1, p < 0.0001] and remained constant until day 14. The interaction of *time* × *treatment* revealed a significant effect for the ipsilateral hind paw of both genotypes [CB2-Syn: F_4,70_ = 27.23, p < 0.0001; CB2-FL: F_4,70_ = 28.63, p < 0.000]. There was no significant *treatment* effect for the contralateral sides of both genotypes [CB2-Syn: F_1,70_ = 0.1315, p = 0.7180; CB2-FL: F_1,70_ = 0.4214, p = 0.5184].

We next plotted the results differently, in order to compare the mechanical allodynia of all four mouse strains directly (Fig. 1D). This analysis showed not only the genotype differences on the contralateral hind paw, but also revealed a genotype effect on the ipsilateral hind paw. Thus, a strong *genotype* effect was revealed by two-way ANOVA for both sides [ipsi: F_3,124_ = 29.35, p < 0.0001; contra: F_3,124_ = 59.21, p < 0.0001]. These results show that CB2KO and CB2-LysM mice not only developed a mirror-image pain phenotype, but also an increased mechanical allodynia in the ipsilateral hind paw, when compared to WT or CB2-Syn mice.

### Induction of a CB2-GFP reporter in myeloid cells after nerve injury

**Figure 2:**
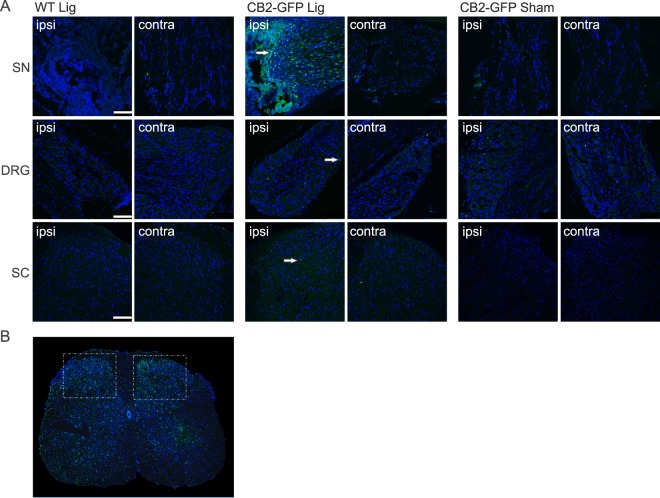
Expression of the CB2 receptor in nervous tissue. (**A**) Representative stainings of GFP (green) in the sciatic nerve (SN), dorsal root ganglia (DRG) and dorsal horn of the lumbar spinal cord (SC). (**B**) Lumbar section of the spinal cord, illustrating both dorsal horns, in a WT animal 14 days after nerve ligation. Iba1 staining (green) is increased on the ipsilateral side (right). White frames indicate imaged areas for the SC sections (**A**). GFP expression under a CB2 promoter is shown in ligated and sham treated CB2-GFP mice 14 days post SNL. Ligated WT animals served as control. Arrows indicate GFP positive cells. Cell nuclei were stained with DAPI (blue) (scale = 100 µm).

Mice expressing GFP under the control of the CB2 receptor promoter (GFP-CB2) were used to visualize CB2-expressing cells^6^ in the sciatic nerve, dorsal root ganglia and lumbar spinal cord after SNL (Fig. 2). Tissues from WT mice served as a negative control.

We found a robust GFP signal in the ipsilateral sciatic nerve (SN) after SNL, whereas dorsal root ganglia (DRG) and the dorsal horns of lumbar spinal cord (SC) displayed a much weaker signal. Very little or no CB2-GFP expression was observed in contralateral tissues or sham treated animals. Thus, CB2 was primarily induced in sciatic nerve at the lesion site.

**Figure 3:**
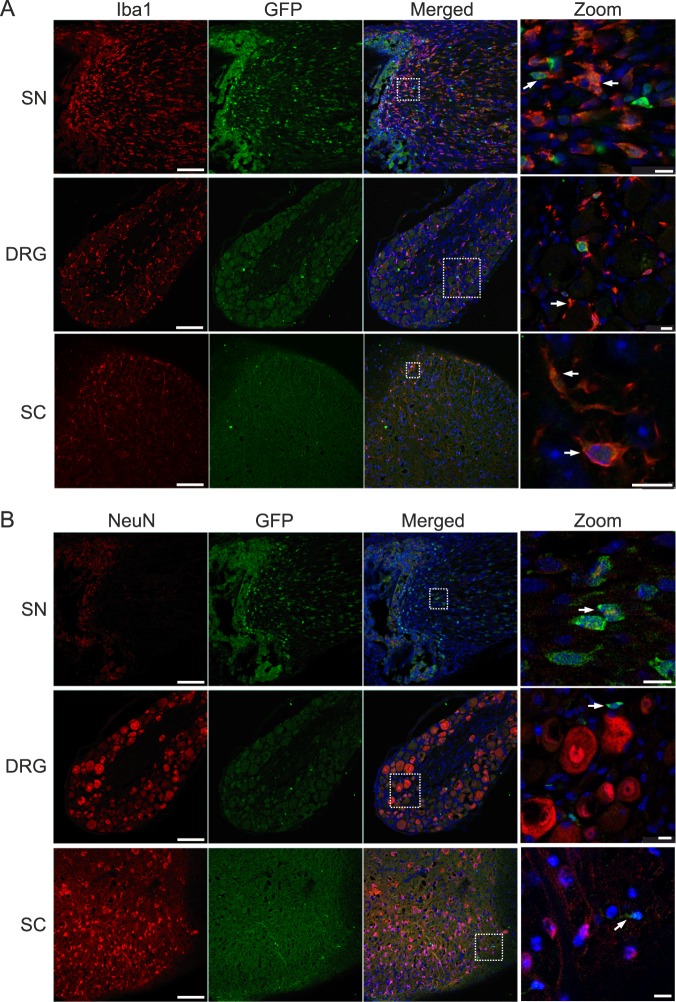
Coexpression of GFP with Iba1 (**A**) and NeuN (**B**). Representative staining of ligated GFP-CB2 14 days post SNL in ipsilateral sciatic nerve (SN), ipsilateral dorsal root ganglion (DRG), and ipsilateral dorsal horns of the lumbar spinal cord (SC). GFP (green) colocalized with Iba1 (red, **A**) but not with NeuN (red, **B**). Arrows show coexpressing cells (scale = 100 µm, zoom scale = 10 µm).

As CB2 is prominently expressed in immune cells, we next co-stained sciatic nerve tissue with Iba1, a marker for macrophages and microglia. Indeed, we observed a distinct CB2-GFP signal in Iba1-positive cells (Fig. 3), strongly suggesting that the CB2-GFP signal originated from myeloid cells at the site of nerve injury. Because sciatic nerve tissue cannot be stained with antibodies directed against the neuronal cell marker NeuN, a nuclear protein, we also investigated the CB2-GFP positive cells in dorsal root ganglia (DRG) and spinal cord tissues. However, CB2-GFP positive cells were not co-labelled with NeuN in these tissues, but with Iba1. These results thus indicate that CB2-GFP is primarily induced after SNL in myeloid cells, but not in neurons.

### Microgliosis in the spinal cord is increased in CB2KO and CB2-LysM mice

**Figure 4:**
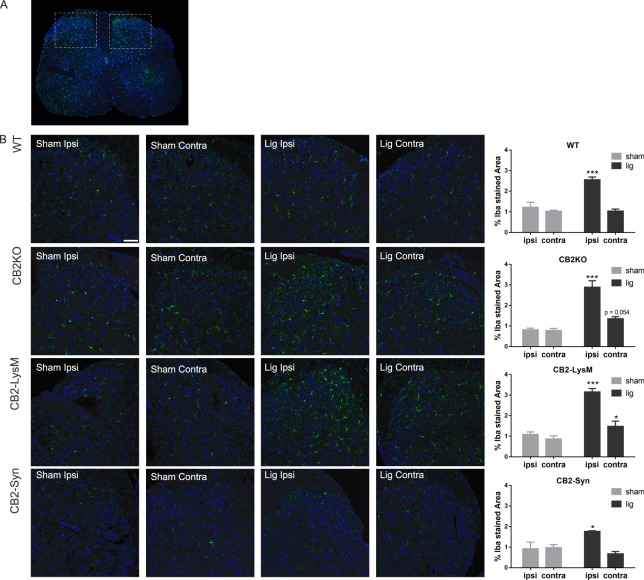
Expression of microglia in the dorsal horn of the lumbar spinal cord. Microglia were stained with Iba1 (green) in both sides of the dorsal horn. (**A**) Lumbar section of the spinal cord. illustrating both dorsal horns, in a WT animal 14 days after nerve ligation. White frames indicate imaged areas for (**B**). (**B**) Representative stainings of Iba1 in CB2KO, WT, CB2-LysM and CB2-Syn mice 14 days after SNL (lig) or sham surgery (sham) (scale = 75 µm). Analysis of Iba1 stained area in percent shows increased staining of Iba1 after ligation ipsilateral (WT, CB2KO and CB2-LysM) and contralateral (CB2KO, CB2-LysM). n = 3–4. Statistical significance between ligated and sham animals for each side and genotype was determined with ANOVA and a post-hoc Holm-Sidak test. Stars represent differences between ligated and sham animals. *p < 0.05, **p < 0.01, ***p < 0.001 Error bars show SEM.

To further investigate the cell-specific deletion of CB2 on the development of neuropathic pain symptoms in CB2KO and CB2-LysM mice, we analysed Iba1 expression as a marker for microgliosis in the dorsal horn of the lumbar spinal cord in WT, CB2KO, CB2-LysM and CB2-Syn mice (Fig. 4). All mice showed a profound increase in Iba1 signal on the ipsilateral side after nerve ligation [WT, p = 0.0008; CB2KO, p = 0.0001; CB2-LysM, p < 0.0001; CB2-Syn, p = 0.0252]. Additionally, CB2-LysM mice displayed an increased signal of the microglia marker Iba1 on the contralateral side [p = 0.0426]. CB2KO animals showed an increase of Iba1 on the contralateral side as well, but failed to induce a statistical significant difference [p = 0.0540]. This was in contrast to WT mice, which did not exhibit an increased contralateral Iba1 signal following nerve ligation [WT, p = 0.9671; CB2-Syn, p = 0.2914]. Sham treated animals did not display any changes in Iba1 stained area in both, ipsi- and contralateral sides of the dorsal horns.

### Leptin receptor expression is increased in the sciatic nerves of CB2-LysM mice

**Figure 5:**
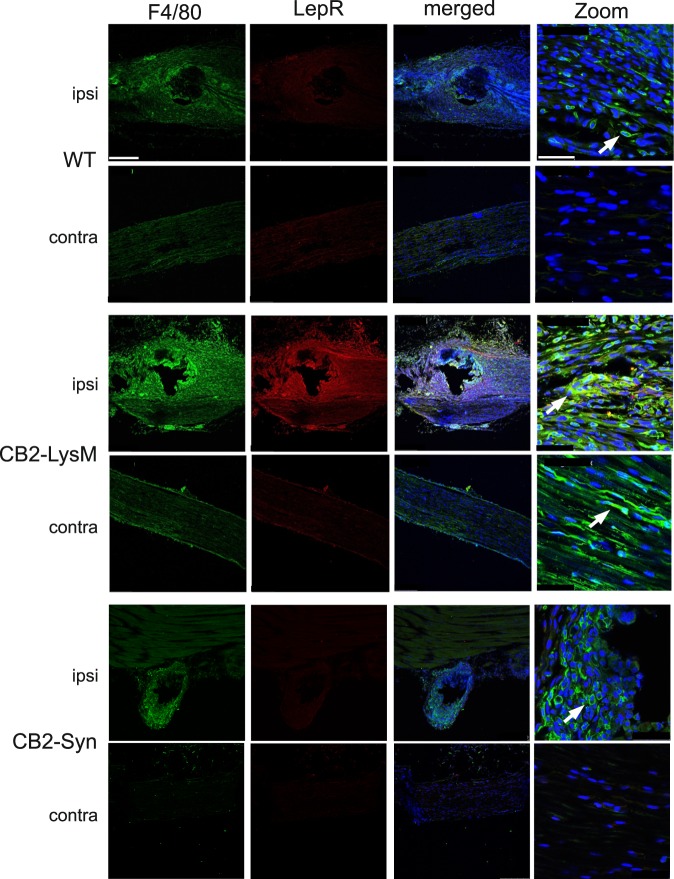
Coexpression of leptin receptor (red) with F4/80 (green). Sciatic nerve tissue of WT, CB2-LysM and CB2-Syn mice was stained 14 days post SNL. Leptin receptor signal was highest and co-labeled with the macrophage marker F4/80 in ipsilateral sciatic nerve of CB2-LysM mice compared to WT or CB2-Syn mice. Arrows show co-labeled cells. (scale = 250 µm, 63x zoom scale = 50 µm).

We have recently shown that leptin signaling is enhanced in CB2KO mice and contributes to the neuropathic pain phenotype in these mice. We now wished to examine if a similar enhancement is also observed in conditional CB2 knock-out mice. We thus analysed leptin receptor (LepR) expression by immunohistochemistry in the ipsi- and contralateral sciatic nerve of WT, CB2-LysM and CB2-Syn mice (Fig. 5). Our results show that LepR expression was enhanced in ipsilateral sciatic nerves of CB2-LysM animals when compared to WT or CB2-Syn mice. This was similar to our previous observation in CB2KO mice. Moreover, LepR co-localised with F4/80, a macrophage marker, indicating that CB2 functions to down-regulate LepR expression in these cells.

## Discussion

In this study, we used different genetic mouse models to demonstrate that CB2 receptors on myeloid cells (macrophages, microglia) modulate neuroinflammatory responses after partial sciatic nerve ligation. Deletion of the CB2 receptor from these cells recapitulates the mirror-image allodynia and increased neuropathic pain phenotype of constitutive CB2 knockouts. In contrast, mice with a deletion of CB2 receptors from neurons show a similar mechanical allodynia and similar neuroinflammation after SNL as WT controls. Thus, although neuronal functions of CB2 have been demonstrated in several paradigms^7^ and although CB2 receptor expression seems to be induced under neuropathic pain condition^8–12^, CB2 receptors on myeloid cells rather than neurons appear to be the main modulators of neuropathic pain, at least in the SNL model used here.

CB2 receptors are notoriously difficult to localize, due to their relatively low expression level in most healthy tissues and the lack of antibodies that work well in tissue sections^32–34^. These difficulties have resulted in a long-standing discussion whether or not CB2 receptors are present in neurons and what functions they may have in these cells. This discussion has also included neuropathic pain conditions, because CB2 receptors were found to be induced on neurons, microglia and satellite ganglion cells after peripheral nerve injury^8^ and because CB2 agonists ameliorate nociceptive pain symptoms^9,16,17,19,20,22,24,25,35–37^.

We have recently generated a bacterial artificial chromosome (BAC) transgenic CB2-GFP reporter mouse strain, in which GFP is expressed under control of the CB2 promotor^6^. This reporter strain re-capitulates the expression pattern of the CB2 receptor and can thus be used as an additional tool to investigate CB2 expression. After SNL, the GFP signal increased, with the highest levels in the ipsilateral ligated sciatic nerve. Co-localization studies with the microglia marker Iba1 and the neuronal marker NeuN showed that the signal originated mostly from microglia and/or macrophages but only negligibly from neurons. We found generally low levels of expression in neuronal tissues, which is in line with other expression studies^4–6^. This finding strongly indicates that the CB2 modulation of neuropathic pain responses is due to its actions on myeloid cells at the side of the nerve injury, rather than remote regions such as DRGs or spinal cord. These findings also agree with previous studies showing CB2 receptor expression in cultured microglia and macrophages, as well as activated monocytes^38^. These results do not support the idea that CB2 protein is also present on sensory neurons in the proximal side of the sciatic nerve^39^, but this negative finding should not be taken as evidence against this possibility. We also detected a GFP signal in cells that were negative for Iba1 and NeuN. These cells could be T lymphocytes, which express CB2 and are known to infiltrate inflamed tissues^40^. It is also possible that the signal originated from ganglion satellite or Schwann cells. It is less likely that the signal is produced by neutrophils, because those are mostly vanished 8 days after nerve injury^41^. Clearly, further studies are necessary to delineate the cellular origin of the reporter gene expression after SNL.

Our results from cell-specific CB2 knockout mice also support a role for this receptor on microglia/macrophages, rather than neurons, in the modulation of neuropathic pain conditions. Thus, neuropathic pain responses in mice lacking CB2 receptors on neurons were indistinguishable from WT controls. We have previously shown that CB2-Syn mice exhibit a reduced power of hippocampal gamma oscillations^7^, thus demonstrating that neuronal CB2 receptors are functionally relevant. The lack of a SNL-induced neuropathic pain phenotype is therefore a distinct indication that neuronal CB2 receptors are not important in the manifestation of neuropathic pain symptoms. It remains to be determined, however, if CB2 receptors modulate the affective component of neuropathic pain, similar to CB1 receptors^42^.

In contrast, CB2-LysM mice recapitulated all aspects of the neuropathic pain phenotype observed in constitutive CB2 knockouts. These included a similar level of mechanical allodynia in the ipsilateral hind paws, which was more pronounced than in controls, a delayed mirror-image allodynia in the contralateral hind paw, enhanced microgliosis of the lumbar spinal cord with less but consistent contralateral inflammation, and an increased expression of LepR in the injured sciatic nerve.

It should be noted that all mouse strains investigated in this study showed an increased Iba1 signal on the ipsilateral side of the nerve injury 14 days after nerve ligation, but only CB2KO and CB2-LysM mice also showed an increased Iba1 signal on the contralateral side. The induction of Iba1 on the ipsilateral side was expected as microglia are known to be essential for the induction and persistence of chronic pain conditions and are known to be increased in the ipsilateral dorsal horn after nerve injury^43^. Signal molecules that activate microglia are released from injured neurons in the spinal cord after peripheral nerve damage, through which the peripheral inflammation is shifted to central sites and develops into a chronic disease^44^. However, it is still unknown how the signal spreads to the contralateral side. The phenomenon of contralateral mirror-image pain has already been described in humans and rodents^27,28^, but the molecular mechanism involved in this phenomenon remain largely obscure. Nevertheless, microglia have been implicated^29,30^. Importantly, an invalidation of CB2 receptors is not a prerequisite for developing mirror-image pain, as most studies on this were conducted in WT mice or rats, without any transgenic modification. To our knowledge, however, mirror-image pain was never observed in WT animals who underwent SNL, but rather in animals after spinal nerve ligation, chronic construction injury or unilateral construction of the infraorbital nerve^45–47^. It is highly plausible that these pain models induce a stronger inflammation than the partial sciatic nerve ligation. We hypothesise that the observed contralateral pain in CB2KO and CB2-LysM mice is a consequence of an increased inflammatory response in the absence of CB2 signaling on myeloid cells.

In conclusion, our paper demonstrates an important contribution of the CB2 receptor on microglia and macrophages, but not on neurons, in the development of neuropathic pain. These results help to clarify the role of the endocannabinoid system in chronic inflammatory pain.

## Methods

### Animals

For the described experiments, constitutive and conditional CB2KO mouse lines were used. Wildtype C57BL/6J (WT) or floxed (CB2-FL) littermates were used as control animals. Experimenters were blind to the animal’s genotype. All animals were bred on a C57BL/6J background. Mice were kept under specific pathogen free conditions (SPF) and were housed with a 24 h light-dark circle (12 h light, 12 h dark). All mice were group-housed in cages with up to five littermates and had *ad libitum* access to food and water. All animals were 2–5 months of age at the time of the experiments.

The CB2KO mice used in our experiments had a deletion in the coding exon of the *Cnr2* gene, thus inactivating the CB2 receptor as described previously^48^. Conditional CB2KO mice were generated using the Cre/loxP recombination system^49^. CB2-FL mice contain two loxP sequences, which flank the open reading frame of exon 2 in the Cnr2 gene^7^. For the generation of conditional KO mice, CB2-FL mice were crossed with mice, expressing the Cre recombinase under cell-specific promoters. CB2-LysM mice resulted from crossing CB2-FL mice with LysM-Cre mice^50^ and have consequently a conditional deletion of Cnr2 in myeloid cells. In CB2-Syn mice, the Cre recombinase is expressed under the synapsin 1 promoter, which is specifically expressed in neurons^7,51^. As a consequence, the Cre recombinase is only active and excises parts of the *Cnr2* gene in neuronal cells. CB2-GFP mice^6^ were used to localize CB2 receptor expression. All experiments followed the guidelines of the German Animal Protection Law and were approved by the Landesamt für Natur, Umwelt und Verbraucherschutz Nordrhein-Westphalen. (AZ 84-02.04.2014.A258, AZ 87-51.04.2014.A393, AZ 84-02.04.2014.A443).

### Partial sciatic nerve ligation (SNL)

To induce a sciatic nerve injury, the partial sciatic nerve ligation (SNL) was used^52^. Mice were anesthetized with 2–3% isoflurane gas and 1/3–2/3 of the left sciatic nerve was tightly ligated with a 7-0 braid silk suture (Natsume Seisakusho, Tokyo, Japan). This resulted in a robust development of mechanical allodynia with its peak on day 14 post ligation. Sham operated mice underwent the surgery without ligation of the sciatic nerve and served as controls. Mechanical allodynia was tested with von Frey filaments (Stoelting. USA) using the up-down method described previously^53^. Mice were habituated on a metal grid for 1 h on three days prior to the first measurement and 1 h directly before each assessment. All mice were tested before and 3, 7, 10 and 14 days after SNL. Von Frey filaments of different force were gently applied to the plantar surface of the hind paws until the filament bent. Shaking, licking or paw withdrawal was considered as a nociceptive response. By varying between different filaments, a value was generated, which could be calculated into the force that generates a response in 50% of all cases^53^.

### Immunohistochemistry (IHC)

For the immunohistological experiments, tissue was collected at 14 days post-surgery using the same procedure. Ligated and sham operated animals were anesthetized with a mixture of 10% ketamine and 5% xylazine. Mice were then intracardially perfused for 5 min with ice-cold phosphate buffered saline (PBS) and 15 min with 4% formaldehyde. The tissue samples (lumbar spinal cord, DRGs, and sciatic nerves) were isolated, post-fixed overnight and cryopreserved in 30% sucrose. Tissue samples were then embedded in O.C.T. compound (Tissue Tek, Sakura, The Netherlands), frozen to −80 °C and sliced into 14 µm thick sections at a cryostat (CM3050S, Leica, Germany). For Iba1 stainings, spinal cord sections were permeabilized 15 min in 0.1% Triton X-100 (Sigma) solved in PBS and blocked for 2 h in 10% normal donkey serum/PBS. Anti-Iba1 antibody (019–19741, Wako, 1:500) was incubated overnight and washed with PBS on the next day. Secondary antibody (A31573, Invitrogen, 1:1000) was incubated for 2 h and washed off with PBS. Slides were then embedded in mounting media (Dapi Fluoromount G, SouthernBiotech, USA), covered and sealed. For leptin IHC, the same protocol with different antibodies was used. Primary antibodies included one against leptin receptor (AF497, R&D Systems, USA, 1:40) and one against F4/80 (CL8940AP, Cedarlane, USA, 1:100) a surface marker for macrophages. Secondary antibodies used were against leptin receptor antibody (705-166-147, Jackson Immuno Research Laboratories, 1:250) and F4/80 antibody (A21208, Life technologies, 1:250). Since the expression of CB2 is generally low and thus GFP is only slightly expressed, we used anti-GFP antibodies to amplify the GFP signal. To analyse GFP in CB2-GFP and WT mice, tissue was stained with an anti-GFP antibody (ab6673, Abcam, 1:1000) in combination with either anti-Iba1 (019–19471, Wako, 1:500) or anti-NeuN (MAB377B, Millipore, 1:200). Before the first antibody incubation, an antigen retrieval step was performed, which consisted of a 40 min long incubation in citrate buffer at 70 °C. Sections were then permeabilized with 0.5% Triton X-100 solved in PBS for 1 h and blocked with 10% normal donkey serum for 2 h. Primary antibodies were incubated overnight and washed off with PBS on the next day. Secondary antibodies against GFP antibody (705-166-147, Jackson Immuno Research Laboratories, 1:1000), Iba1 antibody (A31573, Invitrogen, 1:1000), or NeuN antibody (405207, Biozol, 1:300) were incubated for additional 2 h and washed off with PBS. Slides were then embedded in mounting media (DAPI Fluoromount G, SouthernBiotech, USA). Stained tissue was imaged through a confocal microscope (LSM SP8, DMI 6000 CS, Leica) and analysed with Image J (1.47 v Wayne Rasband, NIH, USA). Image analysis of Iba1 stained spinal cord tissue in Fig. 4 was performed as previously described^26^. In short, the area of Iba1 stained tissue in the dorsal horn of the spinal cord was quantified by thresholding the stained images in ImageJ. The resulting values represented stained Iba1 area in percent and were compared between both treatments within the same genotype. Per genotype, 3–4 animals were analysed with each 3–4 sections of the dorsal horn on each side.

### Data analysis

All data are presented as means ± standard error of the mean (SEM). Data was calculated and analysed by ImageJ (v1.47), Microsoft Excel (Office 2013) or Graphpad Prism (v.6.0c). To calculate statistical significance, two-way ANOVA plus an additional Bonferroni’s post-hoc test or a Dunnet’s post-hoc test was used for the assessment of mechanical allodynia and a Holm-Sidak post-hoc test for the Iba1 analysis. Significance level was set to p ≤ 0.05. Experiments from Figs 1 and 4 were analysed with a post-hoc power analysis, using G*Power 3.1.9.2 (University of Düsseldorf). Results are shown in Table 1.

**Table 1:** Power Analysis.

Figure		groups	side	Power			Effect size (f)		
				time	treatment	interaction	time	treatment	interaction
Fig. 1	a	WT	ipsi	1.0000	1.0000	1.0000	1.1310	2.2064	1.2058
			contra	0.0960	0.2020	0.1000	0.1141	0.1362	0.1186
		CB2KO	ipsi	1.0000	1.0000	1.0000	2.0334	3.8966	2.1440
			contra	1.0000	1.0000	1.0000	1.0306	1.1489	1.2319
	b	CB2-FL	ipsi	1.0000	1.0000	1.0000	1.2546	2.2808	1.2100
			contra	0.4547	0.3621	0.6233	0.3316	0.2153	0.3973
		CB2-LysM	ipsi	1.0000	1.0000	1.0000	1.6015	2.9857	1.8587
			contra	1.0000	0.9999	1.0000	1.2010	0.9756	1.3044
	c	CB2-FL	ipsi	1.0000	1.0000	1.0000	1.2425	2.7622	1.4857
			contra	0.5164	0.1051	0.4159	0.2982	0.0775	0.2652
		CB2-Syn	ipsi	0.9999	1.0000	1.0000	0.9319	2.0504	1.2473
			contra	0.1829	0.0669	0.3668	0.1699	0.0433	0.2483
				**time**	**genotype**	**interaction**	**time**	**genotype**	**interaction**
Fig. 1	d		ipsi	1.0000	1.0000	0.9348	3.0005	0.8426	0.4286
			contra	1.0000	1.0000	1.0000	1.0275	1.1968	1.1298
				**Power**			**Effect size (f)**		
				**side**	**treatment**	**interaction**	**side**	**treatment**	**interaction**
Fig. 4		WT		0.9992	0.9808	0.9776	1.6139	1.2638	1.2433
		CB2KO		1.0000	1.0000	1.0000	4.8548	2.6019	4.6779
		CB2-LysM		0.9998	1.0000	0.9896	1.8442	2.6242	1.4203
		CB2-Syn		0.8381	0.3509	0.9049	0.9727	0.5165	1.0812
